# Error statistics of hidden Markov model and hidden Boltzmann model results

**DOI:** 10.1186/1471-2105-10-212

**Published:** 2009-07-09

**Authors:** Lee A Newberg

**Affiliations:** 1 The Wadsworth Center, New York State Department of Health, Albany, NY 12201, USA; 2 Department of Computer Science, Rensselaer Polytechnic Institute, Troy, NY 12180, USA

## Abstract

**Background:**

Hidden Markov models and hidden Boltzmann models are employed in computational biology and a variety of other scientific fields for a variety of analyses of sequential data. Whether the associated algorithms are used to compute an actual probability or, more generally, an odds ratio or some other score, a frequent requirement is that the error statistics of a given score be known. What is the chance that random data would achieve that score or better? What is the chance that a real signal would achieve a given score threshold?

**Results:**

Here we present a novel general approach to estimating these false positive and true positive rates that is significantly more efficient than are existing general approaches. We validate the technique via an implementation within the HMMER 3.0 package, which scans DNA or protein sequence databases for patterns of interest, using a profile-HMM.

**Conclusion:**

The new approach is faster than general naïve sampling approaches, and more general than other current approaches. It provides an efficient mechanism by which to estimate error statistics for hidden Markov model and hidden Boltzmann model results.

## Background

Hidden Markov models are employed in a wide variety of fields, including speech recognition, econometrics, computer vision, signal processing, cryptanalysis, and computational biology. In speech recognition, hidden Markov models can be used to distinguish one word from another based upon the time series of certain qualities of a sound [1]. In finance, the models can be used to simulate the unknown transitions between low, medium, and high debt default regimes in time [2]. In computer vision they can be used to decode American Sign Language (ASL) [3]. Hidden Markov models are used in computational biology to find similarity between sequences of nucleotides (DNA or RNA) or polypeptides (proteins) [4,5] and to predict protein structure [6].

Hidden Markov models permit the facile description and implementation of powerful statistical models and algorithms that are used for calculation of the *probability *of sequential data. Furthermore, the algorithms used to manipulate hidden Markov models are easily applied more generally. Frequently these dynamic programming algorithms are instead employed in the calculation of an odds ratio, which is the the ratio of the probability of sequential data under a foreground model (signal), divided by the probability of the sequential data under a background model (noise). In other applications, the algorithms are used to compute other scores, frequently employed as proxies for logarithmic probabilities or logarithmic odds ratios, even though the scores are not directly derived from known foreground and background statistical models. Below, we will precisely define a hidden Boltzmann model as a hidden Markov model generalization that admits these odds ratio and other score calculations.

Perhaps the most common use of hidden Boltzmann models is for the purpose of hypothesis testing or classification. For instance, a speech-recognition model may be used to quantify the belief that a sound bite is the word "elephant." However, once a score for a belief has been computed, the question is how to interpret that value.

1. Is the score strong enough to indicate a signal, or is it reasonably probable that noise will yield a score this strong?

2. Is the score weak enough to indicate noise, or is it reasonably probable that a signal will yield a score this weak?

The *false positive rate *(closely related to the *type I error *or *p-value*) for a score threshold is the probability that noise data will yield a score at least as strong as the threshold. The *true positive rate *for a score threshold is the probability that signal data will yield a score at least as strong as the threshold.

Within computational biology, error statistics are used primarily in the subfield of sequence alignment, where specialized approaches exist for computing them. (See the next section.) We are hopeful that the availability of the general approach we describe here will enable the productive use of error statistics in other subfields of computational biology, and in other scientific fields where error statistic estimation has been difficult.

### Prior work

Methods for estimating the false positive rate exist in some settings. For instance, we can consider the Smith-Waterman pairwise local alignment algorithm [7], which can be interpreted as a maximum path score calculation via a hidden Boltzmann model. This well-established algorithm scores the extent to which two sequences have similar subsequences; recent techniques permit efficient estimation of false positive rates for this algorithm to levels as low as 10^-4000 ^[8]. Efficient estimation is also available for the more general local profile-HMM sequence alignments [9].

Furthermore, in the special case where a hidden Boltzmann model computes a logarithmic odds ratio and where the score threshold is not too extreme, there is a generally applicable technique [10]. In this prior work, each probability parameter of a hidden Markov model is modified to be a weighted arithmetic average of applicable background and foreground probabilities, (1 - *α*)*p*_*B *_+ *αp*_*F *_for *α *∈ [0, 1], where *p*_*B *_is the applicable probability under the background model, and *p*_*F *_is the applicable probability under the foreground model. When the score function for a hidden Boltzmann model happens to be a logarithmic odds ratio, the technique we present here can be described similarly. However, under such a circumstance, our modified hidden Boltzmann model has an "unnormalized" probability that is a weighted geometric average of the background and foreground probabilities, *p*_*B*_^1-*α *^*p*_*F*_^*α *^for *α *≥ 0. (Note that even in this limited context of logarithmic odds ratios, we are able to estimate error statistics for higher score thresholds than are achievable in the prior work because, by permitting any *α *≥ 0, we allow an extrapolation beyond the *p*_*F *_value.)

Here we expand and extend the previous false-positive-rate result for pairwise sequence alignments [8] to the class of hidden Boltzmann models, which includes the class of hidden Markov models. In particular, we extend the result to biologically relevant hidden Markov models of all sorts, not just profile-HMMs. We demonstrate the new technique in the Method sections, and we show that the approach is applicable to true positive rates as well. We make use of a novel importance sampling distribution and provide a novel approach to computing its normalization.

In the Results section, we discuss our application of the technique within the HMMER 3.0 package, which permits scanning nucleotide and polypeptide sequence databases for patterns of interest. In particular, we show that it works well with HMMER global alignments.

## Methods

We describe first our models and then the algorithms we use to manipulate them.

### Models

For our building blocks we assume that we are given: a hidden Boltzmann model that computes scores of interest for sequences of interest, a simple *background model *(also termed *null model *or *random model*) that describes *noise *sequences, and a computable *foreground model *(also termed *alternative model *or *hypothesis model*) that describes *signal *sequences. Each of these will be described more thoroughly in the following.

#### Hidden Boltzmann models: states, transitions, and emissions

With little or no modification, many algorithms applicable to hidden Markov models are useful more generally. These algorithms, including the present work, function not only with the strict probabilities of a hidden Markov model, but also with odds ratios, with logarithms of probabilities or logarithms of odds ratios, and with scores used as proxies for such logarithms. Nonetheless, the term "hidden Markov model" is more restrictive and does not admit these generalizations. To accommodate these generalizations, we coin the term "hidden Boltzmann model," so named because of earlier work with Boltzmann chains and Boltzmann machines [11,12]. Much as is done with Boltzmann chains and Boltzmann machines, we describe hidden Boltzmann models in terms of scores rather than probabilities. Although these scores are often scaled logarithmic probabilities or scaled logarithmic odds ratios, in general they need not be. A hidden Boltzmann model consists of a set of *states *and a set of directed *transitions *between states. Any state or any transition can be designated as an *emitter*. Each emitter includes a specification of the set of *emissions *that it can produce; these emissions are from an *alphabet*, the set of all possible emissions. Furthermore, each state, each transition, and each emission of each emitter has a real-valued *score *(also termed *energy*) associated with it.

An *emission path *through a hidden Boltzmann model starts at a special *start *state, ends at a special *terminal *state, proceeds from state to state via transitions, and includes a choice of emission for each encounter with each emitter. The *sequence *associated with an emission path is the ordered set of emissions.

The *score *of an emission path is the sum of the encountered transition, state, and emission scores; each score is included in the sum each time that it is encountered along the emission path. Note that when each of the scores is the scaled logarithm of a probability, the summing of scores along an emission path gives the scaled logarithm of the joint probability of events modeled as statistically independent.

**Figure 1:**
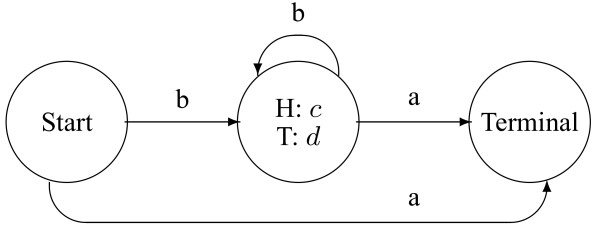
**A simple hidden Boltzmann model**. A hidden Boltzmann model that emits sequences of "H" and "T" characters. The score associated with a particular emitted string is *a *+ (*h *+ *t*)*b *+ *hc *+ *td *where *a*, *b*, *c*, and *d *are real-valued scores, and *h *and *t *are respectively the number of "H" and "T" characters emitted.

As an example, Figure 1 shows a hidden Boltzmann model that emits a string of "H" and "T" characters, modeling the "head" and "tail" results from statistically independent flips of a possibly biased coin. The score associated with a particular emission path is the sum of the encountered scores. For this hidden Boltzmann model the formula for the score of an emission path is easily determined; it is *a *+ (*h *+ *t*)*b *+ *hc *+ *td*, where *a*, *b*, *c*, and *d *are the real-valued scores associated with the transitions and emissions, where *h *is the number of "H" characters emitted and *t *is the number of "T" characters emitted, and where state and transition scores not indicated in the figure are assumed to be zero. Note that hidden Boltzmann models are not restricted to emitting from discrete alphabets, such as the present {H, T}; a hidden Boltzmann model can emit arbitrary real number values, for example. As in the discrete alphabet case, each possible emission for each emitter has an associated score. In this continuous case, such a score is equal to, or is a proxy for, the logarithm of a probability density or the logarithm of the ratio of a foreground probability density to a background probability density.

#### Multiple emission paths for an emitted sequence

**Figure 2:**
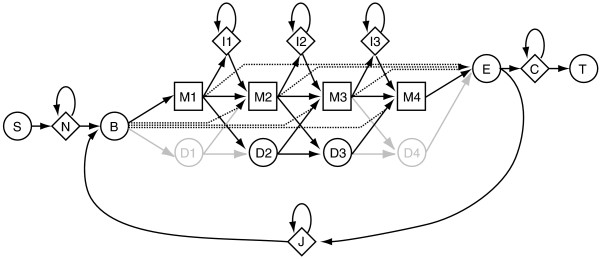
**A Plan7 profile-HMM**. This is the *Plan7 profile-HMM *employed in the HMMER package for scans of nucleotide or polypeptide sequences [13]. Most transitions are assigned scores (not shown). Additionally, each match state (M) and each insertion state (I) emits a character, as does each of the self-loop transitions for the prefix (N), suffx (C), and joining (J) states. Typically, the emission scores vary among the match states; they can vary among the insertion states as well. A score of zero is employed for each possible emission from the N, C, and J self-loop transitions. The D1 and D4 states are shaded, to indicate that, unlike the other positions, the first and last positions of a profile-HMM do not have delete states (D).

We say that the Boltzmann models are *hidden *because, in most cases, an underlying emission path cannot be uniquely determined from its sequence of emissions. In other words, a given sequence can typically be emitted by any of several emission paths through a hidden Boltzmann model, although that is not the case for the simple model of Figure 1. In this more general case (see Figure 2, a HMMER Plan7 profile-HMM [13]), the score associated with an emission sequence is usually determined in either one of two ways, *maximum score *(also termed *Viterbi score*) or *forward score *[1,4].

For a sequence *D*, the maximum score *s*_max_(*D*) is the largest score achievable by an emission path that emits the sequence *D*:

(1)

where *π *∈ *π*_*D *_indicates that any emission path *π *that emits *D *should be considered, and where *s*(*π*) is the sum of all state, transition, and emission scores encountered on the emission path *p*. Despite the usually combinatorially large number of emission paths *π *∈ *π*_*D*_, the value *s*_max_(*D*) is efficiently computable by the standard Viterbi dynamic programming algorithm.

The definition of the forward score *s*_fw_(*D*) for a sequence *D *reflects a hidden Markov model interpretation of the hidden Boltzmann model. For any transition, state, or emission score *s *from the hidden Boltzmann model, the value exp(*s*) is treated as if it were a corresponding hidden Markov model probability, even though generally it is not actually a probability. (For instance, these values do not behave as probabilities in that, for any given state of a hidden Boltzmann model, the outgoing transition exp(*s*) values need not sum to one.) Because an emission path's score *s*(*π*) is computed as the sum of the scores encountered as it is traversed, exp(*s*(*π*)) is interpreted as the product of the encountered probabilities. Furthermore, exp(*s*_fw_(*D*)) is computed as if it were the overall probability of an emitted sequence *D*, where distinct emission paths through the model are assumed to be statistically disjoint events:

(2)

The name *forward *comes from the algorithm used to calculate this sum. The algorithm has run-time and memory efficiency comparable to those for the corresponding *s*_max_(*D*) algorithm [1,4].

A third approach for combining scores across emission paths corresponds to the definition of free energy from thermodynamics. The *partition function Z*(*D, T*) and corresponding *free score s*_free_(*D, T*) for any *temperature T *∈ (0, +∞) are

(3)

Note that exp(*s*_free_(*D, T*)/*T*) can be computed via a minor modification to the forward algorithm that computes exp(*s*_fw_(*D*)); it is the values of exp(*s/T*), exp(*s*(*π*)/*T*), and exp(*s*_free_(*D*)/*T*) that are treated as if they were hidden Markov model probabilities in the forward algorithm. The run-time and memory efficiency for the *s*_free_(*D*, *T*) computation are essentially the same as those for the *s*_fw_(*D*) or *s*_max_(*D*) computation. We will make use of this partition function in the following.

#### The background model

We assume a simple background model for sequences of a specified length *L*. Specifically, we assume that under a background model *B*, the *L *sequence positions are statistically independent and identically distributed according to some shared probability distribution Pr(*d|B*), where *d *indicates a possible emission:

(4)

where *d*_*i *_is the *i*th emission of the sequence *D*. This assumption might be relaxed; see *Complex background models *in the Discussion section.

#### Mathematical problem statement

The score for a sequence *D *of length *L *is compared to other sequences of the same length. We write

(5)

where the false positive rate fpr(*s*_0_) that we wish to estimate is the probability-weighted fraction of background model sequences of length *L *that achieve a score of at least *s*_0_, where *D *∈ *D*_*L *_indicates that any sequence *D *of length *L *should be considered, where Pr(*D|B*) is the probability of a sequence *D *under the background model, where *s*(*D*) is the score assigned to the sequence *D *by the hidden Boltzmann model, and where Θ is a function that has value one if its argument is true or value zero if its argument is false. We write *s*(*D*) to indicate that this definition applies to *s*(*D*) = *s*_max_(*D*) and to *s*(*D*) = *s*_fw_(*D*).

### Algorithm

#### Importance sampling

The error statistic estimation algorithm is a simulation via importance sampling. Although exhaustive computation of the sum in Equation 5 is usually not feasible, the value of fpr(*s*_0_) can be estimated via naïve sampling. That is, sequences are sampled/generated according to the background model *B*, and fpr(*s*_0_) is estimated by the fraction of the sampled sequences with a score of at least *s*_0_. We note that if Pr(*D|T*) is the probability of a sequence *D *under some other model for sequences of length *L *that is parameterized by a value *T*, then we can write

(6)

Where

(7)

We can estimate the value of fpr(*s*_0_) by sampling sequences according to this alternate model, and then averaging the corresponding *f *(*D, s*_0_) values. This approach is called *importance sampling *[14]. Importance sampling is useful because estimation via Equation 6 can be substantially more efficient than estimation via Equation 5. That is, in terms of the variances of the the estimators, often it is possible to find an importance sampling model for which

(8)

#### Choice of importance sampling distribution

We define the importance sampling model parameterized by *T *as

(9)

where *Z*(*T*) is the normalization of the Pr(*D|T*) probability distribution and is defined as

(10)

Insertion of this definition for Pr(*D|T*) into Equation 7 gives

(11)

The value that we will choose for the parameter *T *∈ (0, +∞) has yet to be specified.

#### Importance samples

Ultimately we wish to draw sample sequences according to the distribution Pr(*D|T*), compute *f *(*D*, *s*_0_) for each sample, and use the average of these values as our estimate for the false positive rate. Here we describe the sampling of sequences.

Employing the background model specified by Equation 4, we compute the value *Z*(*T*) via a novel modification to the forward algorithm that computes *Z*(*D*, *T*). In the forward calculation of *Z*(*D*, *T*), the emission of a value *d *from an emitter *E *is incorporated via a factor exp(*s*_*E*_(*d*)/*T*), where *s*_*E *_(*d*) is the score associated with the emission of *d *from the emitter *E*. In the forward calculation for *Z*(*T*), instead of such a factor we use the average factor for the emitter ⟨exp(*s*_*E*_/*T*)⟩_*B*_,

(12)

regardless of the value of *d*. Because the needed pre-computation and caching of these average factors are typically significantly faster than is the forward score computation, the run time for the *Z*(*T*) calculation is essentially the same as that for the *Z*(*D*, *T*) or *s*(*D*) computations.

We sample a sequence of length *L *via stochastic backtrace of the *Z*(*T*) forward computation. Specifically, we sample the states and transitions of an emission path *π *from the *Z*(*T*) computation via standard hidden Markov model techniques for stochastic backtrace [1,4,15]. In addition, we sample emissions for the emission path, where the probability that a value *d' *is sampled for an encounter with an emitter *E *is

(13)

Thus, we have sampled *p *(*i.e*., its states, transitions, and emissions) from the probability distribution

(14)

We then disregard the sampled states and transitions, retaining only the sampled emissions, a sequence *D*. Because the sequence *D *could have arisen from any emission path *π *that emits it, the probability that we will sample *D *by this approach is

(15)

which is the promised importance sampling distribution of Equation 9.

#### Estimation of the false positive rate

We wish to estimate the false positive rate for a threshold *s*_0_, for either maximum scores or forward scores depending upon the application. For each of *N *sampled sequences {*D*_*i*_: *i *= 1 ... *N*}, we compute *s*(*D*_*i*_) and *Z*(*D*_*i*_, *T*). An estimate for fpr(*s*_0_) is then

(16)

where  is an estimate of the statistical true negative rate. Alternatively, we can estimate fpr(*s*_0_) with

(17)

There are additional alternatives, such as

(18)

and

(19)

In our implementation for HMMER 3.0 (see the Results section) we have found  to work well. The choice for the best estimator usually depends upon the efficiency of the estimators, which can be estimated from the *N *importance sampled sequences.

#### Estimation of the true positive rate

The technique for the estimation of false positive rates can be extended to the estimation of true positive rates or, equivalently, false negative rates. We can modify the above technique to estimate

(20)

where the true positive rate tpr(*s*_0_) is the probability-weighted fraction of foreground model sequences of length *L *that achieve a score of at least *s*_0_, where Pr(*D|F*) is the probability of a sequence *D *of length *L *under the foreground model *F*, and where fnr(*s*_0_) is the false negative rate. The importance sampling estimate derives from the relationship

(21)

Where

(22)

(23)

#### Special case for the true positive rate

Equation 23 simplifies further under a common scenario. For this special case, we assume that the scores of the hidden Boltzmann model are logarithmic odds ratios built from some foreground hidden Markov model *H *and the background model *B*, and that the foreground model *F *is the restriction of the model *H *to sequences of a length *L*:

(24)

(25)

In Equation 24, *D*(*π*) is the sequence emitted by the emission path *π*. Use of Equation 24 in Equation 3 and in Equation 10 gives

(26)

(27)

Therefore, in this special case:

(28)

Thus, two estimators for the true positive rate are

(29)

And

(30)

We can define the estimators  and  in a manner analogous to the definitions of the estimators  and  in Equations 18 and 19. Note that when we are estimating the true positive rate for a *forward *score threshold, we already have *Z*(*D*_*i*_, 1) in hand; it is equal to exp(*s*_fw_(*D*_*i*_)) and *s*_fw_(*D*_*i*_) is needed for the computation of Θ(*s*(*D*_*i*_) ≥ *s*_0_).

#### Choice of temperature

To complete the the Algorithm section, we need an approach to select a temperature *T *that will be efficient for a given score threshold *s*_0_. Because the relationship between temperature and score threshold is not straightforward, we recommend the building of a calibration curve to relate temperature to maximum score, and the building of a second calibration curve to relate temperature to forward score. Furthermore, for both maximum scores and forward scores, we have empirically observed lower variances for error statistic estimation when the fraction of sampled sequences exceeding the given score threshold is 20–60%; thus, we recommend aiming for a value for the importance sampling temperature parameter that achieves this statistic.

Conceptually, the approach to building a calibration curve is straightforward. For each of several temperatures, compute *Z*(*T*) and perform several stochastic backtraces to sample several sequences from the Pr(*D|T*) distribution. For each sampled sequence *D*, compute its score *s*(*D*). Plot the resulting temperature-score pairs {(*T*_*i*_, *s*_*i*_)} as points in the *x*-*y *plane. Via some reasonable *ad hoc *procedure, use such a plot to choose a temperature for each score threshold of interest. Once a temperature is selected, draw and process *N *importance samples to compute the error statistics, as previously described.

## Results

Using the alpha-release source code for the HMMER 3.0 package [9], we randomly generated a length *M *= 100, Plan7 profile-HMM, and we estimated its error statistics for local-alignment scans of polypeptide sequences of length *L *= 200. Our calibration curves had 50 temperatures, and required 100 calculations of *s*_max_(*D*) for the maximum score threshold calibration curve and 100 calculations of *s*_fw_(*D*) for the forward score threshold calibration curve. We used the appropriate calibration curve to choose a temperature for each score threshold that we subsequently considered. For each of 1000 maximum score thresholds and for each of 1000 forward score thresholds, we estimated the false positive and true positive rates.

For each forward score threshold, we used *N *= 100 calculations of *s*_fw_(*D*), 100 calculations of *Z *(*D*, *T*), and 1 calculation of *Z*(*T*) to estimate the false positive rate, and an additional 1 calculation of *Z*(1) to estimate the true positive rate. Similarly, for each maximum score threshold, we used 100 calculations of *s*_max_(*D*), 100 calculations of *Z*(*D*, *T*), and 1 calculation of *Z*(*T*) to estimate the false positive rate, and an additional 100 calculations of *s*_fw_(*D*) and 1 calculation of *Z *(1) to estimate the true positive rate. Because all other parts of the error statistic calculations require comparatively little run time, the calculation of both error statistics for a specified forward or maximum score threshold required 202–302 times the run time of a typical *s*(*D*) calculation. The error statistics calculation for a score threshold is 4.2–6.3 seconds on our platform. Note, however, that considerable savings in run time could have been achieved through the selective re-use of samples from one score threshold for another; see *Re-use of simulations *in the Discussion section.

**Figure 3:**
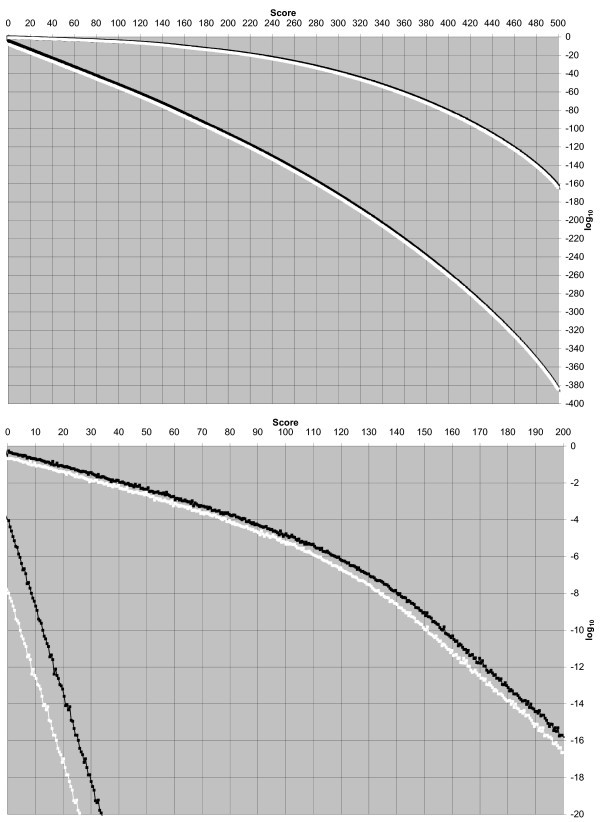
**False positive rate and true positive rate plotted against score threshold**. This figure is demonstrative of the ease by which error statistics estimates can be had and demonstrates low-score-linear and high-score-concave regions. The bottom panel depicts an enlargement of the upper left corner of the top panel. In both panels, from top to bottom the curves are (1) forward score true positive rate, (2) maximum score true positive rate, (3) forward score false positive rate, and (4) maximum score false positive rate. For example, for a score threshold of 100, the maximum score false positive rate is 10^-55 ^and the forward score false positive rate is 10^-51^; for this threshold, the maximum score true positive rate is 10^-5.2 ^and the forward score true positive rate is 10^-4.8^. The low-score-linear and high-score-concave regions of the *false positive rate *curves are qualitatively as expected, based upon the Gumbel distribution approximation and its break down, respectively. For the *true positive rate *curves, the demonstration of low-score linearity and the bend/phase transition near the score of 125 may be novel. Despite the extreme statistics, the values for these plots are easily computed; we employed *N *= 1000 importance samples for each of 1000 maximum score thresholds and each of 1000 forward score thresholds.

The run-time for a naïve sampling approach for any of these computations would be significantly larger, on the order of 0.02 seconds divided by the computed error statistic; an error statistic less than 10^-20 ^would require a run-time longer than the present age of the universe. Special purpose approaches, such as that for profile-HMM local sequence alignments, are typically faster than the importance sampling approach. Computed false positive rates and true positive rates, as a function of score threshold, are plotted in Figure 3. See the Discussion section.

Previously, we applied the algorithm to real DNA sequences; we employed the approach to analyze Smith-Waterman pairwise local alignments of intergenic regions in five Drosophila species, and easily estimated false positive rates as low as 10^-400 ^[16].

## Discussion

We have provided a technique for the error statistic estimation of hidden Boltzmann model results. For all but the lowest hidden Boltzmann model scores, the presented technique is significantly more efficient than naïve simulation. We have demonstrated the effectiveness of the technique in the HMMER 3.0 package for scanning sequence databases.

### Review of results

#### Applicability to hidden Markov models

The approach for the general class of hidden Boltzmann models is easily specialized to hidden Markov models. The natural logarithm of any transition or emission probability *p *of a hidden Markov model is used in lieu of the corresponding score *s *in a hidden Boltzmann model. In particular, in the above formulae any occurrence of exp(*s*) should be replaced with *p*, and any occurrence of exp(*s/T*) should be replaced with *p*^1/*T *^.

#### Linearity of error statistics as a function of score threshold

The lowermost two curves plotted in each panel of Figure 3, for maximum score and forward score *false positive rates*, are relatively straight for false positive rates above 10^-100^. This behavior is consistent with theory and observations that these curves represent Gumbel distributions [9,17]. Also as expected, the curves bend downward as the scores become more extreme. This is an indication that the Gumbel distribution result, which applies asymptotically as sequence lengths increase without bound, breaks down for extreme scores. This break down has been observed before [8,18] and is expected [19]: in short, whether for maximum score or forward score, a highest achievable score exists among sequences of a fixed length *L*, and the curves will go to a false positive rate of zero as that score is approached.

The two uppermost curves in each panel of Figure 3, for maximum score and forward score *true positive rates*, are linear for scores under 100, and bend downward for more extreme scores. We are unaware as to whether this low-score linear behavior has been observed or predicted previously. Unlike the false positive rate curves, these curves experience a "phase transition," near a score of 125; the slope of the curves changes and then enters another linear regime. The cause of this phase transition merits further exploration.

#### Non-extreme error statistics

In our experience, importance sampling is more efficient than naïve sampling for false positive rates under 10^-6^. For higher values, especially above 10^-3^, the relationship of Equation 8 breaks down, and naïve sampling is often more efficient. Furthermore, the scores that yield these false positive rates also demark the transition in relative efficiency for true positive rate estimation.

### Future directions

#### Real problem instances

A significant shortcoming of the present work is our insufficient testing on real problem instances. Except for the special case of Smith-Waterman local DNA alignments [16], this hidden Boltzmann model technique has not been tested on real data. In future work we anticipate demonstrating the effectiveness of the present work on scans of actual protein and nucleotide databases, using accepted hidden Boltzmann models that are designed to identify common evolutionary history and/or common functionality. Such testing has been important in prior work [9,20].

#### Scaling to different problem instances

In past work, for Smith-Waterman local sequence alignments, we have noted that the logarithmic false positive rate curves, such as those depicted in Figure 3, are remarkably conserved in shape [8]. That is, we have observed that an affine transformation of a logarithmic false positive rate curve for sequences of some length *L*_1 _is a remarkably good approximation of the corresponding curve for sequences of some other length *L*_2_. Furthermore, the needed affine transformation is easy to calculate without simulations; it is the unique transformation that takes both the (minimum score, maximum logarithmic false positive rate) point and the (maximum score, minimum logarithmic false positive rate) point for sequences of length *L*_1 _to the corresponding points for sequences of length *L*_2_.

The extent to which conservation of shape applies to the general class of hidden Boltzmann model error statistic curves is a topic that merits further consideration.

#### Re-use of simulations

When the score thresholds in a set of interest are not too different from one another, a single temperature and a single set of *N *sampled sequences can be used to calculate the error statistics for the entire set of score thresholds. The calculations of *s*(*D*), *Z*(*D*, *T*) and *Z*(*D*, 1) are the most time-intensive part of the error statistics calculations, but they need be performed only once for each sampled sequence. Therefore, the error statistics for a set of nearby score thresholds can be estimated almost as quickly as they can be estimated for a single threshold. In particular, if error statistics are needed for a large number of score thresholds, it will be productive to cache all computed *s*(*D*), *Z*(*D*, *T*) and *Z*(*D*, 1) values at each employed temperature, for possible use with subsequent score thresholds. However, because the efficiency of the error statistic estimators depends significantly upon the choice of temperature, use of samples from a given temperature *T *should be avoided unless 20–60% of the samples for that temperature satisfy *s*(*D*) ≥ *s*_0_. While a run time equal to a few hundred score calculations is much shorter than is achievable by previous techniques, it is still undesirably slow for many applications, including HMMER 3.0. Importance sample caching and error statistic curve scaling will help to bring down the overall run time required for multiple error statistic estimations.

#### Other scoring functions

Other definitions of score exist. For example, a definition that corresponds to the thermodynamic concept of average energy is

(31)

We expect that the techniques presented here will successfully carry over to free score, average score, and other definitions of score.

#### Complex background models

A modification of Equation 23, which is for estimating true positive rates under an arbitrary *foreground *model, might yield efficient estimation of false positive rates under a complex *background *model.

Specifically, if the background model  is more complex than as indicated by Equation 4, but is sufficiently approximated by a model *B *that does satisfy Equation 4 then

(32)

(together with Equations 6, 3, and 10) prescribes an importance sampling approach for computing false positive rates under the complex background model. Under what circumstances this approach will be efficient is an open question.

#### Stochastic context-free grammars

The present technique can be applied to the Inside/Outside algorithms that manipulate stochastic context-free grammars [21]; much as we have described here, use of a *p*^1/*T *^value in lieu of each probability *p *in a grammar gives an unnormalized probability distribution that can be used for importance sampling. We conjecture that the resulting importance sampling distribution will lead to significantly more efficient estimation than naïve sampling. In computational biology, stochastic context-free grammars are used with RNA secondary structure [4,22], though we have not seen statistical significance estimation in this context.

## Conclusion

We have demonstrated a technique for error statistic estimation for hidden Boltzmann models and shown how it is applied to hidden Markov models. The approach is faster than naïve sampling approaches and is more general than other current approaches.

## Authors' contributions

LAN conceived and designed the experiments, performed the experiments, analyzed the data, and drafted the manuscript.
